# The Role of the Popeye Domain Containing Gene Family in Organ Homeostasis

**DOI:** 10.3390/cells8121594

**Published:** 2019-12-07

**Authors:** Johanna Ndamwena Amunjela, Alexander H. Swan, Thomas Brand

**Affiliations:** 1 Department of Cell and Molecular Biology, St. Jude Children’s Research Hospital, Memphis, TN 38105, USA; Johanna.Amunjela@stjude.org; 2 Institute of Chemical Biology, Department of Chemistry, Imperial College London, London W12 0BZ, UK; a.swan17@imperial.ac.uk; 3 Developmental Dynamics, National Heart and Lung Institute (NHLI), Imperial College London, Imperial Center of Translational and Experimental Medicine, London W12 0NN, UK

**Keywords:** cyclic adenosine monophosphate (cAMP), signaling, membrane trafficking, cardiac arrhythmia, muscular dystrophy, adhesion, cancer, cell proliferation, migration, invasion

## Abstract

The Popeye domain containing (POPDC) gene family consists of *POPDC1* (also known as *BVES)*, *POPDC2* and *POPDC3* and encodes a novel class of cyclic adenosine monophosphate (cAMP) effector proteins. Despite first reports of their isolation and initial characterization at the protein level dating back 20 years, only recently major advances in defining their biological functions and disease association have been made. Loss-of-function experiments in mice and zebrafish established an important role in skeletal muscle regeneration, heart rhythm control and stress signaling. Patients suffering from muscular dystrophy and atrioventricular block were found to carry missense and nonsense mutations in either of the three POPDC genes, which suggests an important function in the control of striated muscle homeostasis. However, POPDC genes are also expressed in a number of epithelial cells and function as tumor suppressor genes involved in the control of epithelial structure, tight junction formation and signaling. Suppression of *POPDC* genes enhances tumor cell proliferation, migration, invasion and metastasis in a variety of human cancers, thus promoting a malignant phenotype. Moreover, downregulation of *POPDC1* and *POPDC3* expression in different cancer types has been associated with poor prognosis. However, high *POPDC3* expression has also been correlated to poor clinical prognosis in head and neck squamous cell carcinoma, suggesting that *POPDC3* potentially plays different roles in the progression of different types of cancer. Interestingly, a gain of *POPDC1* function in tumor cells inhibits cell proliferation, migration and invasion thereby reducing malignancy. Furthermore, POPDC proteins have been implicated in the control of cell cycle genes and epidermal growth factor and Wnt signaling. Work in tumor cell lines suggest that cyclic nucleotide binding may also be important in epithelial cells. Thus, POPDC proteins have a prominent role in tissue homeostasis and cellular signaling in both epithelia and striated muscle.

## 1. Introduction

Cyclic adenosine monophosphate (cAMP) is a second messenger that relays cellular signals downstream of G-protein coupled receptors (GPCRs), to regulate multiple targets that trigger cellular responses to various ligands [1,2,3]. Signaling of the cAMP pathway is initiated when a ligand binds to its binding site on a G-protein coupled receptor (GPCR) causing the activation of G_α_s. G_α_s stimulates adenylyl cyclase (AC), which converts adenosine triphosphate (ATP) to cAMP [2,4]. At elevated intracellular cAMP levels, cAMP binds to effector molecules to regulate multiple processes downstream of the pathway.

There are five known cAMP effector proteins in mammalian cells: protein kinase A (PKA), exchange protein directly activated by cAMP (EPAC), Popeye domain containing (POPDC) proteins, cyclic nucleotide receptor involved in sperm function (CRIS) and the cyclic nucleotide–gated ion (CNG) channels [5]. PKA is the most extensively studied cAMP-binding protein and for many decades, it was the only known effector protein [6]. However, EPAC and the CNG channels were subsequently discovered to be also regulated by cAMP and to play important roles either by cooperating with PKA or independently mediating signaling events. The identification of these effector proteins established that cAMP has a much wider range of roles and regulates multiple signal transduction pathways and cellular responses. More recently, CRIS and the POPDC protein family were identified as novel classes of cAMP binding molecules, further expanding the number of cAMP effector proteins [5,7,8]. The roles of cAMP in regulating physiological processes are therefore broad, as cAMP serves as a second messenger to facilitate signal transduction downstream of a diverse multitude of GPCRs.

Although all five cAMP-binding proteins facilitate cAMP-mediated signaling to regulate cell behavior, this review focuses specifically on the role of the POPDC proteins in regulating normal physiology and their dysregulation in various pathologies.

## 2. The POPDC Protein Family

The Popeye domain containing (POPDC) proteins are a novel class of cAMP binding, transmembrane proteins. The POPDC gene family consists of three isoforms: *POPDC1, POPDC2* and *POPDC3* [2,4,9]. *POPDC1*, also known as blood vessel epicardial substance (*BVES*), was first discovered 20 years ago. *POPDC1* was initially named *BVES* due to its observed expression in epicardial and coronary vascular cells, while the name Popeye genes was given due to the strong expression in striated muscle cells [1,8]. The paralogues *POPDC2* and *POPDC3* were subsequently discovered and also encode transmembrane proteins carrying an intracellular Popeye domain. Hence the three genes form a family named after the Popeye domain, which is shared by the three proteins.

**Figure 1:**
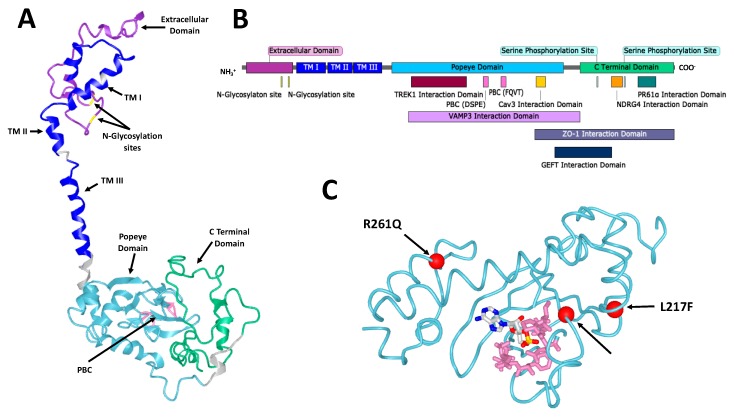
The structure of Popeye domain containing (POPDC) proteins. (**A**) A homology model of human POPDC1. POPDC1 shares a similar structure with POPDC2 and POPDC3. Some features are indicated including the extracellular domain (purple), the two Asn residues of the N-glycosylation sites (yellow), the three transmembrane (TM) domains (blue), the Popeye domain (cyan), the DSPE and FQVT motifs, which are part of the phosphate binding cassette (PBC, pink) and the C-terminal domain (green). The model was produced using the *Phyre2* algorithm [16]. (**B**) A linear map of POPDC1. Structural features are indicated as well as the sites of interaction of multiple interaction partners. Many of the interaction sites are approximate and have not been precisely identified. (**C**) A homology model of the Popeye domain of human POPDC3, shown with cyclic adenosine monophosphate (cAMP) in its predicted binding site. The DSPE and FQVT motifs of the PBC are shown in pink. The positions of the three pathological mutations in *POPDC3* reported by Vissing et al. [17] are shown as red spheres. The model was produced using the *Phyre2* algorithm and the cAMP binding site was predicted using the 3DLigandSite predictor [16,18].

The structure of POPDC proteins consists of a short (27–39 residues) extracellular amino terminus followed by three transmembrane domains, a cytoplasmic Popeye domain and the carboxyl terminal domain (CTD), which is of variable length and the sequence is isoform-specific [2,7] (Figure 1). The protein is tethered to the plasma membrane as a dimer that in the case of POPDC1 is stabilized by a disulfide bridge. The Popeye domain serves as a specific high-affinity binding site for cAMP [10].

The POPDC isoforms share the same protein structure but differ in protein size. POPDC2 is the largest of the three isoforms consisting of 367 amino acids, while POPDC1 is 359 amino acids long and POPDC3 is the smallest isoform containing only 292 amino acids [11]. The size difference is mainly determined by the length of the CTD.

Interestingly, the extracellular N-terminus of POPDC1 harbors two N-linked glycosylation sites at Asn^20^ and Asn^27^ [12]. It is, however, unclear whether N-linked glycosylation affects POPDC1 function or its ability to interact with tight junction and cell adhesion molecules. Furthermore, it is not yet known whether POPDC1 contains any O-linked glycosylation sites. Alterations in the N- and O-glycosylation status, and in glycan branching patterns of transmembrane proteins are known to compromise cell–cell adhesion and promote cell invasion and metastasis [13]. Alterations in the glycosylation patterns of POPDC1 at its extracellular glycosylation sites could thus potentially affect its function and ability to interact with tight junctions and adhesion molecules. In this regard, it is noteworthy that the molecular weight of POPDC1 found in heart muscle has a molecular weight of 58 kDa, while in skeletal muscle or brain, the predominant protein species has a molecular weight of 70 kDa, suggesting tissue-specific glycosylation might be of some functional importance [14,15]. Further studies are warranted to explore the importance of N- and O-linked glycosylation on POPDC1 function.

## 3. POPDC Proteins as cAMP Effector Proteins

The binding mode of cAMP has been predicted through modelling and mutagenesis studies as no empirical structure of the cyclic nucleotide binding domain (CNBD), or full-length POPDC protein, has been reported [19]. There is, however, a lack of sequence homology to the classical cAMP effector proteins such as PKA and EPAC, with the closest match being that of bacterial catabolite activator (CAP) or cAMP response (CRP) proteins with approximately 20% homology. CRP or CAP proteins are transcription factors, which bind DNA as dimers and their DNA binding affinity is enhanced upon cAMP binding [20]. Secondary structure predictions of the Popeye domain identified similarities to a CNBD [19]. A homology model of the Popeye domain was built using the CNBD structure of CRP of *Streptomyces coelicolor* (PDB: 2PQQ) as a template and the CNBD of PKA RII (PDB: 1CX4) for further structure refinement [19]. The Popeye domain sequence is highly conserved across the three isoforms and present in all species from hydra to man [11]. Two ultra-conserved motifs are present in the Popeye domain, DSPE and FQVT, which were predicted by the homology model to be directly involved in cAMP binding and were assigned the role of a putative phosphate binding cassette (PBC) [19] (Figure 1). The Popeye domain’s PBC is, again, highly divergent from canonical PBCs found in other CNBDs [21]. Confirmation of cAMP binding was achieved through affinity precipitation and radio-ligand binding studies. cAMP inhibited ^3^H-cAMP binding to the Popeye domain with an IC_50_ of around 118nM, which is comparable to the cAMP binding affinity of the CNBD of PKA. An approximate 40-fold selectivity for cAMP over cGMP was also demonstrated. A series of point mutants altering the sequence of the PBC of POPDC1 was created to examine the importance of the predicted PBC. A D200A variant showed a 90% loss of cAMP affinity, while a S201F mutation, which was discovered in patients suffering from limb-girdle muscular dystrophy displayed a 50% reduction in cAMP binding affinity [14]. E203A and V217A showed modest reductions in affinity compared to wild type. In contrast, although proline 202 is strongly conserved, the P202A mutation caused no difference in cAMP affinity [19].

### 3.1. Mutations in POPDC1 and POPDC3 Affect cAMP Binding or cAMP Responsiveness

The uncertainty in the binding mode of cAMP to the Popeye domain, and the functional changes this induces within the protein and downstream, means that understanding the importance of cAMP binding in the known POPDC mutations is challenging. Nevertheless, changes in the binding of cAMP to the Popeye domain have been suggested to be important and causally involved in the pathologies observed in carriers of POPDC mutations. For example, the POPDC1^S201F^ variant shows a 50% reduction in cAMP binding affinity and homozygous carriers of this mutation suffer from limb-girdle muscular dystrophy (LGMD) and cardiac arrhythmia [14]. An important finding was that when co-expressed with the TWIK1-related K^+^ channel (TREK1), a two-pore-domain background potassium channel, in *Xenopus* oocytes, POPDC1 increases outward K^+^ currents, which is further enhanced in the case of the POPDC1^S201F^ mutant [14]. Interestingly, incubating *Xenopus* oocytes in media containing 1mM 8-Br-cAMP abolished this effect in the wild type but had no effect on the mutant. This suggested that the loss of cAMP-controlled modulation of TREK1 currents was in part responsible for the cardiac arrhythmias observed in patients possessing the POPDC1^S201F^ mutation and corresponding animal models [14].

However, insensitivity to cAMP binding seems to be unable to explain the effect of all known POPDC family mutations. The three recently reported pathological mutations in *POPDC3* (L155H, L217F and R261Q), which were discovered in patients suffering from limb-girdle muscular dystrophy show variability in their effect on cAMP binding [17] (Figure 1). A repeat of the co-expression experiment with TREK1 in *Xenopus* oocytes was run. It showed wild type POPDC3 reduces TREK1 current compared to TREK1 expressed alone, with the mutants having varying effects. R261Q partially restored TREK1 current while both L155H and L217F increased currents relative to those seen for TREK1 alone. As with POPDC1, raising cAMP concentrations, here with theophylline, abolished the change in TREK1 current induced by wild type POPDC3. While treatment was ineffectual on dampening the effect of the L155H mutant, both the L217F and R261Q mutations were sensitive to cAMP, with theophylline restoring TREK1 currents so as to be insignificantly different from those observed for TREK1 alone. This demonstrates that the L217F and R261Q mutants were able to sense cAMP and exert functional effects on TREK1, while the L155H mutation did not. However, all three mutations caused similar LGMD pathologies, suggesting that potentially different pathogenic pathways may lead to a similar clinical outcome. Whether these mutations directly affect cAMP binding or instead cAMP-induced conformational changes in the protein is not known. These mutants are not part of the putative PBC of POPDC3, but the overall poor understanding of Popeye domain structure makes their effects hard to predict.

### 3.2. Working Models

#### 3.2.1. Switch Model

In absence of a full understanding, attempts have been made to propose working models to describe the role of POPDC proteins in the cAMP pathway. The switch model describes the POPDC proteins as a means through which the effects (activation or inhibition) on interaction partners can be acutely mediated through cAMP signaling. The interaction with TREK1 has provided some evidence for this model [14,19]. While co-expression of POPDC1 and TREK1 increases TREK1 surface expression, in the presence of raised cAMP levels, TREK1 current is not affected by POPDC1. This suggests that POPDC1 is carrying out some form of cAMP-dependent gating role. This is supported by the finding that the interaction between POPDC1 and TREK1 undergoes an acute change upon an increase of cAMP concentration, as reported by a bimolecular FRET assay. However, a 10-min incubation of cells co-expressing POPDC1 and TREK1 with 8-Br-cAMP failed to produce a significant change in TREK1 current [14]. It would be of interest to repeat this experiment with a cAMP analogue with higher membrane permeability or instead forskolin to directly stimulate AC activation.

#### 3.2.2. Shield Model

A further development of the switch model is the shield model. This postulates that as well as cAMP binding acting as a stimulus for changes in protein–protein interactions, phosphorylation of POPDC proteins downstream of β-adrenergic signaling also has functional effects. While phosphorylation of POPDC proteins has been reported, its functional significance has yet to be determined [22]. This may provide some form of feedback mechanism within the cAMP pathway, with POPDC being a target for cAMP-mediated phosphorylation via PKA and other kinases. Moreover, interaction of POPDC proteins with interacting proteins may shield these from being phosphorylated.

#### 3.2.3. Trafficking or Cargo Model

The trafficking model has been proposed based upon the change in interaction partner localization upon POPDC knockout or mutation. Disruption of cAMP binding in the POPDC1^S201F^ mutant led to alterations in TREK1 surface expression when co-expressed in Xenopus oocytes [21]. It is also of great interest that tissues of patients carrying a mutation in *POPDC1* show aberrant membrane localization of POPDC2 [14,23]. As well as indicating the importance of oligomerization in the POPDC family, it suggests a role for cAMP binding in the control of POPDC-mediated protein localization. Further evidence comes from the fact that POPDC1 has been confirmed to directly interact with the vesicular transport protein VAMP3 [24]. Loss of POPDC1 disrupts VAMP3 mediated vesicular transport in MDCK epithelial cells through inhibition of transferrin and β1-intergrin movement.

#### 3.2.4. Sponge Model

There is a continued debate on how local cAMP concentrations are controlled to form cAMP nanodomains. While phosphodiesterases (PDEs) have been shown to play a key role through spatially controlled degradation of cAMP, it has been suggested that some form of cAMP buffering, resulting in a reduced rate of cAMP diffusion, is required to create the observed cAMP nanodomains [25]. The POPDC proteins have been suggested as candidates for such a buffering mechanism. This is supported by their high affinity for cAMP, their high levels of expression and their interactions with proteins in the cAMP pathway. This is known as the sponge model. No direct experimental evidence supporting this model has yet been reported, although the cardiac phenotypes seen in *Popdc1* and *Popdc2* null mutant mice could be interpreted as a hypersensitivity to β-adrenergic signaling, perhaps due to unregulated cAMP diffusion in the cell [19].

It is likely that the role of the POPDC proteins are both isoform- and tissue-specific. Detailed studies will be needed to fully elucidate the subtleties of their role(s) in modulating cAMP signaling. Many of the observed roles of the POPDC proteins have yet to be directly linked with their cAMP binding function. The majority of the protein–protein interactions reported, which seem to be implicated in a range of physiological functions, have not had their sensitivity to cAMP binding examined, the main exception being TREK1.

## 4. The Role of POPDC Proteins in Striated Muscle

POPDC genes are expressed at high levels in the heart and skeletal muscle [11]. While *Popdc1* is expressed at nearly equal levels in both types of striated muscle, *Popdc2* is strongly expressed in the heart and only weakly in skeletal muscle, while the reverse is true for *Popdc3* [26]. In order to gain insight into the function of POPDC genes, null mutations for *Popdc1* and *Popdc2* were generated in mice [19,27]. Homozygous null mutants for both genes are viable and do not display any overt pathology other than lower heart and body weights and an elevated blood pressure [28]. The expression level of *Popdc1* and *Popdc2* in the cardiac conduction system is higher than in the working myocardium. Likewise, the sinoatrial (SAN) and atrioventricular nodes (AVN) show a prominent expression of both genes [19]. In order to test whether the null mutants display a cardiac arrhythmia phenotype, telemetric ECG devices were implanted into mutant and control mice and heart rate and ECG pattern were monitored. Both *Popdc1* and *Popdc2* KO mice develop a stress-induced bradycardia in an age-dependent manner [19]. The bradycardia phenotype is associated with some morphological aberrations such as a loss of cell extensions normally present in pacemaker myocytes [29] and a loss of pacemaker myocytes in the tail region of the SAN [19]. Whether these aberrant morphologies have any functional consequences is, however, presently unknown. Cardiac arrhythmia is also observed in zebrafish *popdc1* and *popdc2* morphants and the *popdc1* null mutant [14,30]. However, in contrast to the sinus bradycardia present in mutant mice, an AV-block is seen in zebrafish.

Recently, mutations in *POPDC1*, *POPDC2* and *POPDC3* have been discovered in patients that suffer from heart and skeletal muscle disease (Table 1). In the case of *POPDC1*, patients that carry mutations develop LGMD and an AV-block of varying degree [14,23]. In contrast, patients carrying a *POPDC2* mutation develop a severe third-degree AV-block, but muscle appears to be normal [31]. The reverse is true for *POPDC3*, as in this case only skeletal muscle is affected and patients develop a severe LGMD, but the heart is normal [17]. The differential effect of POPDC mutations on heart and skeletal muscle maybe related to the expression level of each isoform, or alternatively that the different isoforms have unique functions, specific to cardiac or skeletal muscle, respectively.

In patients carrying *POPDC1* mutations, the expression and subcellular localization of POPDC1 and POPDC2 was studied in skeletal muscle biopsies. Interestingly, membrane localization of the mutant POPDC1 protein was severely compromised and a massive loss of the mutant protein was observed [14,23]. In the case of patients carrying the *POPDC1^S201F^* mutation, a perinuclear expression domain of the mutant protein has been described [14]. For some of the mutations, a reduction in *POPDC1* mRNA through nonsense-mediated decay (NMD) was demonstrated [23]. Unexpectedly, in addition to the aberrant cytosolic localization of POPDC1, the expression of POPDC2 was also significantly impaired. A strongly reduced cytosolic localization of POPDC2 was observed in all patients carrying missense or nonsense mutations of *POPDC1* [14,23]. Interestingly, while *POPDC3* mutations have also been linked to LGMD, the biopsies of patients carrying any one of the three identified POPDC3 mutations do not display aberrant membrane localization of the mutant protein, nor were there any alterations observed in POPDC1 or POPDC2 [17] suggesting that differences exist between the pathogenic processes that are trigged by different POPDC isoforms.

A defect in skeletal muscle structure and function in animal models has so far been best documented in the case of *popdc1* and *popdc2* morphants in zebrafish, which display an aberrant structure of the facial and tail musculature [14,30]. A common feature of the phenotype in both morphants and mutants is the aberrant structure of the myotendinous junction (MTJ), which is a specialized basement membrane and required to transmit force between tendon and muscle [32]. Electron microscopy of the *popdc1* mutants revealed a lack of the electron dense matrix proteins (mainly a network of collagen type, I, III and IV), which accumulates in the MTJ [14]. As a putative consequence of the impaired MTJ development, myofiber detachment was observed in *popdc1*, *popdc2* and, although rare, also in *popdc3* morphants [14,17,30]. The common phenotype seen in case of *popdc1–3* morphants suggests an important role of POPDC proteins in MTJ formation. Since POPDC1 has been demonstrated to interact with dystrophin, which has an important role in MTJ formation in zebrafish, it could potentially define a molecular pathway that is affected by the loss of POPDC protein function [14].

Interestingly, patients carrying *POPDC1* and *POPDC3* mutations display elevated to high CK levels, which suggest compromised sarcolemmal integrity. Minor defects of the plasma membrane are repaired via a mechanism that involves a large number of proteins including dysferlin [33], which was recently identified as an POPDC1-interacting protein [14]. Skeletal muscle fibers of the oldest patient carrying the homozygous POPDC1^S201F^ mutation display focal damage of the sarcolemma, which suggests that impaired sarcolemmal repair may be a feature of carriers of *POPDC1* mutations [14]. Presently it is unclear how POPDC proteins might be involved in membrane repair, which is known to be triggered by elevated cytosolic Ca^2+^-levels. However, Ca^2+^-influx may trigger cAMP production via the activation of a Ca^2+^-inducible AC isoform. Increases in cAMP have been implicated in membrane repair response in the case of some cell types but has not been studied in relation to sarcolemmal repair in striated muscle [34].

Severe muscle injuries involve the activation of satellite cells, a stem cell population, which is located adjacent to the muscle fiber and shares the same basement membrane [35]. Activated satellite cells proliferate in response to injury, fuse to form myotubes, differentiate and increase in size by hypertrophy. Ultimately the newly regenerated muscle fibers are able to substitute the damaged ones and fully restore the contractile function of the injured muscle. Injury of hindlimb muscles of *Popdc1* null mutant mice was experimentally induced by cardiotoxin injection, which triggers Ca^2+^-overload and fiber necrosis [27]. In the *Popdc1* null mutant muscle, regeneration is retarded compared to wildtype mice and newly formed muscle fibers were much smaller in the mutant muscle. The molecular basis for the impaired regenerative response is poorly understood. However, in activated satellite cells, POPDC1 is located in the nucleoplasm, while myotube formation triggers a loss of nuclear localization [36]. It will be interesting to find out whether nuclear function of POPDC proteins involves transcriptional control, given that the bacterial CAP and CRP proteins are the closest related proteins. POPDC proteins apparently have multiple modes to maintain homeostasis in striated muscle.

## 5. The Roles of POPDC Proteins in Cell Proliferation

Although the roles of POPDC genes in the modulation of cell proliferation have not been extensively studied, POPDC1 has been shown to interact with multiple molecules in pathways that regulate cell proliferation. Furthermore, POPDC1 has been shown to affect cell proliferation in various cancer types (Table 2). POPDC proteins have long been thought to function as tumor suppressors that are dysregulated to promote malignant cell behavior such as enhanced cell proliferation, migration and invasion in various cancers [7,37,38,39,40]. However, recent evidence suggests the roles of POPDC genes in cancer might be isoform-specific. POPDC1 is thought to function as a tumor suppressor that inhibits or regulates cell proliferation in normal physiology. To date, evidence on the effects of POPDC1 on cell proliferation and its interaction with molecules in pathways that regulate cell proliferation such as Zonula occludens-1 (ZO-1), Wnt and c-Myc, are consistent with POPDC1 being a regulator of cell proliferation and its loss or suppression of function enhancing malignant cell behavior [7,10,41,42]. The loss of POPDC1 function has further been shown to enhance cell migration, proliferation, and invasion in many solid tumors [10,37,38,42,43,44]. Furthermore, *POPDC1* expression is significantly suppressed at all clinical stages of breast [43] and gastric cancer [45] without correlation to clinical progression. Indeed, the findings from these studies support the hypothesis that POPDC1 is a tumor suppressor of multiple cancer types. However, the mechanisms by which POPDC1 regulates cell growth remain unclear.

POPDC1-mediated inhibition of cell proliferation could be activated downstream of cAMP. At high intracellular levels, cAMP has been shown to promote apoptosis and inhibit cell adhesion, migration and invasion in breast and colon cancer cell lines [46,47].

Elevated intracellular cAMP levels also inhibited tumor growth in mouse xenografts of human colon cancer and breast cancer [48]. These data suggest that the overall effect of cAMP in breast and colon cancer is anti-tumorigenic and is consistent with the observed POPDC1-mediated inhibition of cell migration, proliferation and invasion in various cancer types. The anti-tumorigenic effects of cAMP could therefore be at least partially mediated via POPDC1 [37]. Consistent with this hypothesis, cAMP upregulates the expression of *POPDC1* in breast cancer cells [37], which likely leads to an inhibition of cell migration and proliferation. This hypothesis is further supported by the fact that the other known cAMP binding molecules that affect tumor cell behavior, PKA and EPAC, have been shown to promote malignant behavior in breast cancer. The PKA RI subunit is overexpressed in breast cancer and its overexpression is associated with accelerated cell growth, metastasis, poor clinical outcomes and anti-hormone therapy resistance [49,50]. Furthermore, the suppression of PKA RI inhibits growth and induces apoptosis in breast cancer cells [50,51]. The effects of forced expression of PKA and EPAC1 in breast cancer has also been investigated [52].

**Table 2:** Effects of POPDC proteins on cell proliferation and cell death.

Cell Type	Effects of POPDC Protein on Proliferation	Potential/Suggested Mechanism	Ref.
Colorectal carcinoma cells	Forced expression of POPDC1 reduces cell proliferation	Unknown	[39]
Corneal epithelial cells	POPDC1 affects signaling pathways relevant to cell proliferation	Regulation of RhoA and Wnt signaling	[39]
Epithelial breast cancer cells	Suppression of POPDC1 enhances and forced expression reduces cell proliferation	Not suggested	[37]
Epithelial breast cancer cells	Forced expression of POPDC1 inhibits EGF-mediated cell proliferation	EGF suppresses POPDC1 expression	[43]
Colitis-associated cancer cells	POPDC1 null mutant mice display increased tumor multiplicity	Increased c-Myc levels via POPDC1- PR61α interaction	[10]
Cardiac myocytes	Serum starvation enhances POPDC1 which is protective against apoptosis	Regulation of Rac1 and Bnip3	[53]
Uveal melanoma cells	Forced expression of POPDC1 inhibits cell proliferation	Regulation of ZO-1 and ZONAB	[42]
Mouse embryonic fibroblasts	POPDC1 regulates the activity of pathways relevant to cell proliferation	Interaction with GEFT Regulation of Rac1 and Cdc42	[54]

In addition, pharmacological inhibition of EPAC1 inhibited cell growth and migration in breast cancer. Given that cAMP inhibits growth in breast cancer cell lines and tissues, while EPAC and PKA promote these effects, it is unlikely that cAMP-mediated inhibition of breast cancer cell migration is mediated via EPAC and PKA. However, cAMP-mediated inhibition of breast cancer cell adhesion, migration and proliferation are consistent with the effects seen after forced expression of POPDC1 in breast cancer cells [37,43]. It is thus likely that the anti-tumorigenic effects of cAMP are mediated via POPDC1.

In contrast to POPDC1, POPDC2 and POPDC3, have been associated with both oncogenic and tumor suppressor roles in different cancer types suggesting that these proteins might serve tissue-specific functions. In a recent study that examined the expression of POPDC proteins in ductal breast carcinoma, *POPDC2* and *POPDC3* were found to be significantly overexpressed in breast cancer tissues [43]. Enhanced expression of *POPDC2* was observed at all clinical stages of breast cancer while *POPDC3* was only expressed at higher levels in early stages of the disease [43]. Overall, this data suggests potential oncogenic roles of *POPDC2* and *POPDC3* in breast cancer with *POPDC3* potentially only involved in initiating breast tumorigenesis, while POPDC2 seems to be involved in initiating and sustaining breast cancer at all clinical stages. In breast cancer, HER2+ status correlates to poor prognosis, drug resistance and more aggressive tumors [55,56]. Significant overexpression of *POPDC2* and *POPDC3* were observed in HER2+ tumors suggesting a positive correlation between HER2+ status and the expression levels of *POPDC2* and *POPDC3* [43].

The potential oncogenic functions of *POPDC3* were further demonstrated in head and neck squamous cell carcinoma (HNSCC) where high *POPDC3* expression levels correlated with poor patient survival [57]. In addition, an upregulation of *POPDC3* was also reported in radioresistant esophageal and lung cancer, suggesting that potentially *POPDC3* serves a role in acquired radiotherapy resistance [57].

However, the suppression of *POPDC3* has also been linked to oncogenic functions in gastric cancer. In a study that assessed *POPDC3* expression in gastric cancer tissues, reduced expression levels of *POPDC3* correlated with the depth of invasion, regional lymph node and distant metastasis as well as poor prognosis [40]. Although this study only established a correlation between *POPDC3* expression and cancer progression, causality between loss of *POPDC3* function and enhanced tumor cell migration has also been established. *POPDC3* knockdown was shown to significantly increase cell migration and invasion in epithelial gastric cancer cells [45]. Furthermore, the epigenetic inactivation of *POPDC3* via promoter hypermethylation has also been reported in gastric cancer tissues. Hypermethylation of the *POPDC3* promoter is thus thought to be a potential mechanism by which long-term *POPDC3* suppression is maintained in gastric cancer [45]. Interestingly, in the mouse pyloric epithelium, a specific expression of *Popdc2* has been observed [58]. However, an association of *POPDC2* with gastric cancer such as adenocarcinoma still awaits future investigations.

Given that *POPDC3* demonstrates potential tumor suppressor roles in gastric cancer while also correlating with oncogenic roles in breast cancer and head and neck carcinoma, the effects of *POPDC3* are likely to be tissue-specific. Uncovering the molecular mechanism that underly the divergence in POPDC3 function in different tissues will provide better insight on how the protein could be targeted in different cancers. This suggests that the roles of *POPDC2* and *POPDC3* might differ in different cancers while POPDC1 potentially regulates cell proliferation in a similar manner in different tissues. 

Different functions for the three POPDC isoforms are also suggested by the isoform-specific expression pattern, which has been observed for each of the three family members [26]. While high expression levels in striated muscle tissue is shared amongst the POPDC gene family, the expression in non-muscle tissue is highly divergent and isoform-specific, which corroborates the notion, that there might be tissue-specific functions.

**Figure 2:**
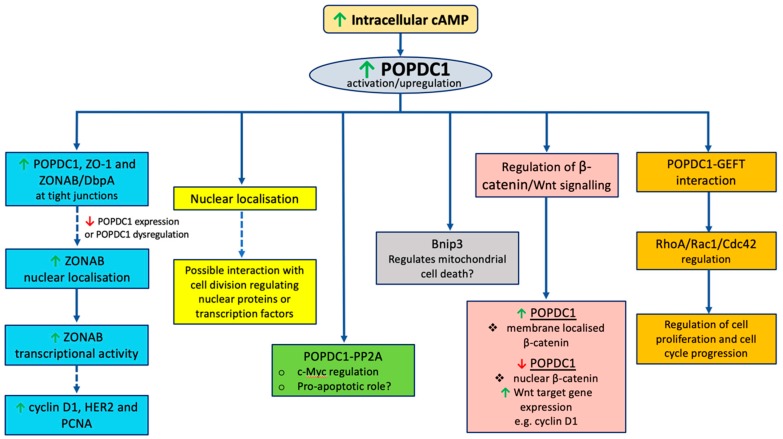
Proposed mechanisms by which POPDC1 may modulate cell proliferation. High intracellular levels of cAMP upregulate the expression of POPDC1. At high expression levels, POPDC1 interacts with multiple partners to suppress cell proliferation. ↑ represents upregulation of POPDC1 expression, ↓ represents downregulation of POPDC1 expression.

The mechanisms by which POPDC proteins regulate cell growth remain unclear. The following section explores potential mechanisms by which POPDC proteins could affect cell proliferation. Given that most research linking POPDC proteins to cell proliferation has been conducted on POPDC1, the mechanisms discussed below are based on validated POPDC protein interaction partners, with a major focus on POPDC1 (Figure 2).

### 5.1. Nuclear Localization and the Interaction with Zonula Occludens 1 (ZO-1)

POPDC1 is found both at the nuclear envelope and in some cell types also in the nucleosplasm. The nuclear localization has been reported in various cell lines including cardiac myocytes, myoblasts and epithelial breast cancer cells [2,9,36]. Furthermore, similar nuclear expression patterns have also been observed in the case of POPDC2 and POPDC3 [36,59,60]. However, the functional implications of this nuclear localization and the mechanisms by which POPDC proteins are transported into the nuclear envelope and the nucleus are currently unknown. Translocation of POPDC1 to the nucleus could play a role in the mechanisms by which POPDC1 regulates cell proliferation such as the potential regulation of cell cycle genes or interaction with nuclear proteins that regulate cell proliferation. We will next discuss the various potential mechanisms by which nuclear localization of POPDC1 could potentially regulate cell proliferation.

Firstly, POPDC1 has been shown to interact with zonula occludens 1 (ZO-1) and to regulate the activity of zonula occludens 1-associated nucleic acid-binding protein/DNA-binding protein A (ZONAB/DbpA) [44,61]. ZO-1 is a tight junction adaptor protein that is known to interact with actin, claudins, occludins, α-catenin and other tight junction proteins including POPDC1 [42,62,63]. Furthermore, ZO-1 has been associated with the regulation of epithelial cell proliferation through ZONAB/DbpA [62,63,64]. The exact mechanism by which ZO-1 regulates cell proliferation is unclear.

However, high ZO-1 expression correlates with decreased cell proliferation and weak nuclear localization of ZONAB/DbpA [65]. ZONAB/DbpA is a Y-box transcription factor that binds to the SH3 domain of ZO-1 to enable its recruitment to tight junctions [62,66]. ZONAB/DbpA has been shown to regulate the expression of proliferative genes and cell cycle regulating genes including HER2, cyclins D1 and PCNA [63,65,67]. Interestingly, when nuclear expression of ZONAB/DbpA is high, epithelial cell proliferation is enhanced [64,65]. However, when ZONAB/DbpA is recruited to the tight junctions by ZO-1, the transcriptional function is suppressed [44,66,68].

The induction of cell proliferation requires high nuclear levels of ZONAB/DbpA and low nuclear expression of ZO-1. What is more, POPDC1 interacts with ZO-1 and high POPDC1 expression correlates with high ZO-1 expression [39,61]. These findings suggest that proliferation is inhibited when POPDC1 and ZO-1 are expressed at high levels. This is further corroborated by the observed suppression of POPDC1 and ZO-1 in various types of cancer cells [37,43,69], consistent with the notion that POPDC1 is a tumor suppressor whose suppression as is the case in some cancer cells enhances cell proliferation.

**Figure 3:**
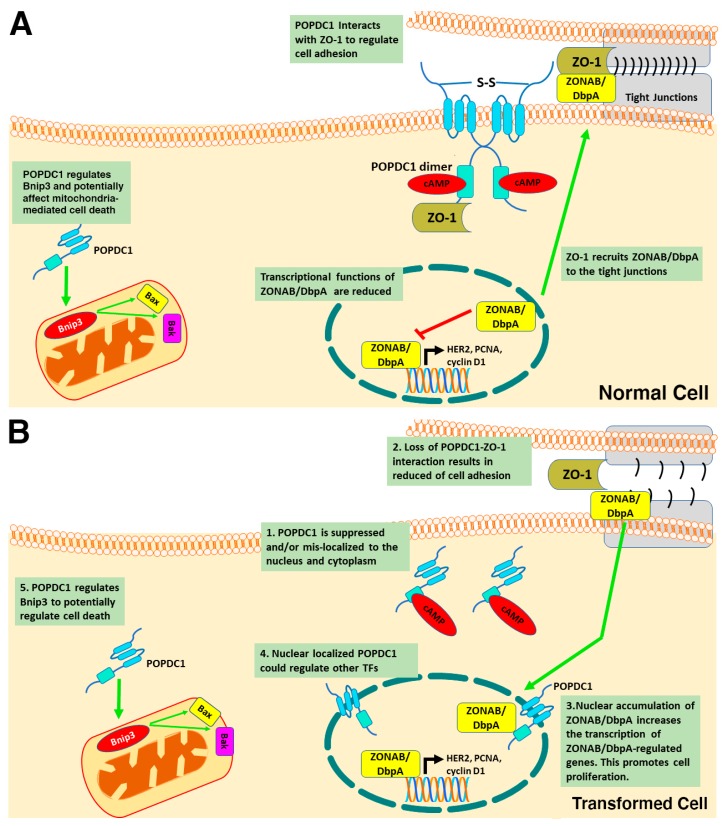
**POPDC1-mediated regulation of cell proliferation in normal and transformed epithelial cells.** (**A**) POPDC1 inhibits cell proliferation in healthy cells. At high intracellular cAMP levels, cAMP binds to the Popeye domain of POPDC1 and upregulates its expression. POPDC1 interacts with ZO-1 at the tight junctions to maintain cell adhesion and inhibit cell migration. ZO-1 recruits ZONAB/DbpA to the tight junctions, suppressing the transcriptional activity of ZONAB/DbpA and inhibiting cell proliferation. POPDC1 also interacts with Bnip3 to suppress mitochondria-mediated apoptosis. (**B**) The dysregulation of POPDC1 in transformed cells affects the cell behavior. 1. Reduction of POPDC1 expression at the cell membrane and increased cytoplasmic and nuclear envelope localization. 2. The interaction between ZO-1 and POPDC1 is lost at the tight junctions resulting in loss of cell–cell contact and enhanced cell migration. 3. ZONAB/DbpA accumulates in the nucleus resulting in increased transcription of ZONAB/DbpA-regulated genes such as HER2, cyclin D1 and PCNA. This consequently enhances cell proliferation. 4. Nuclear localized POPDC1 could potentially regulate the function of other transcription factors to affect cell proliferation. 5. POPDC1 regulates Bnip3 to affect cell death. It is unclear whether this mechanism is dysregulated in transformed cells.

A potential mechanism by which POPDC1 inhibits cell proliferation could thus entail POPDC1 interacting with ZO-1 to inhibit ZONAB/DbpA nuclear localization and consequently preventing the transcription of ZONAB/DbpA-regulated proliferative genes such as cyclin D1, HER2 and PCNA (Figure 3A). This hypothesis would account for the inhibition of cell proliferation in cells with high expression levels of POPDC1 and ZO-1. Furthermore, forced expression of truncated POPDC1 construct, which lacks part of the Popeye domain and the CTD has been observed to increase the intracellular localization and transcriptional activity of ZONAB/DbpA [21] thereby supporting the hypothesis that the dysregulation of POPDC1 potentially promotes cell proliferation via a mechanism that, at least in part, entails enhanced ZONAB/DbpA transcriptional activation of cell cycle regulatory genes (Figure 3B).

### 5.2. The POPDC1–GEFT Interaction

The interaction between POPDC1 and the guanine nucleotide exchange factor (GEFT) presents another potential mechanism by which POPDC1 could regulate cell proliferation. GEFT is a guanine nucleotide exchange factor that modulates the active state of the Ras homologous (Rho) GTPases by exchanging guanosine diphosphate (GDP) for guanosine triphosphate (GTP) [7,70,71]. Interestingly, GEFT is known to activate GTPases that affect cell proliferation such as RhoA, cell division control protein 42 (Cdc42) and Ras-Related C3 Botulinum Toxin Substrate 1 (Rac1) [7,70]. Moreover, GEFT has been shown to interact with the CTD of POPDC1 in mouse embryonic cells and colocalization of the two proteins has also been detected in murine cardiac, skeletal and smooth muscle cells [54]. Furthermore, POPDC1 has been shown to regulate the activity of Rac1 and Cdc42 where forced expression of POPDC1 reduced Rac1 and Cdc42 activity, without affecting RhoA activity [54]. In addition, the exogeneous expression of POPDC1 also increased cell motility [54]. The GTPases Rac1 and Cdc42 are known to function as signaling switches in the regulation of cell proliferation, cell cycle progression, cell–cell adhesion and cell migration [47,72,73,74]. The POPDC1–GEFT interactions could thus potentially serve to regulate cell behavior such as migration via regulation of the activity of GTPases such as Rac1 and Cdc42. Although the effects of the POPDC1–GEFT interaction on cell proliferation have not been explored, POPDC1 could also potentially regulate cell proliferation and cell cycle progression via the regulation of Rac1 and Cdc42 activity. In addition, the observed suppression of Rac1 and Cdc42 activity and increase in cell migration in response to forced *POPDC1* expression in mouse embryonic fibroblasts [54], are consistent with the observed increased tumor cell migration and proliferation in cancer cells where *POPDC1* expression is suppressed [37,44]. Similar data have also been obtained in hepatocellular carcinoma cells (HCC) [38].

Taken together, these findings support the hypothesis that the effects of POPDC1 on cell migration and proliferation could, at least in part, involve GEFT-mediated regulation of Rac1 and Cdc42. However, more studies are required to explore this hypothesis and determine whether endogenous POPDC1 interacts with GEFT in epithelial cancer cells and striated muscle cells where endogenous POPDC1 activity has been observed.

### 5.3. POPDC1, β-catenin and Wnt Signaling

The Wnt signaling pathway is an evolutionarily conserved pathway that plays essential roles during development and in the maintenance of normal physiological function [75]. Activation of the Wnt pathway regulates multiple cellular processes including cell proliferation, cell cycle arrest, apoptosis and tissue homeostasis [76,77]. Dysregulation of Wnt signaling is thus a driving force of tumorigenesis [7,10]. Activation of the canonical Wnt signaling pathway (also known as the Wnt/β-catenin pathway) causes the cytoplasmic accumulation of β-catenin and its subsequent translocation into the nucleus [76,77]. In the nucleus, β-catenin associates with the T-cell factor/lymphoid enhancer-binding factor (TCF/LEF) transcription factors to coactivate the transcription of Wnt target genes such as *CCND1* (cyclin D1) and *JUN* (c-Jun) [10,76,77].

Interestingly, POPDC1 has been shown to modulate the subcellular localization of β-catenin and the transcriptional activity of Wnt target genes [7,44]. POPDC1 interacts with the WNT co-receptor LRP-6 and controls its phosphorylation level by recruiting protein phosphatase 2A (PP2A) [78]. Expression and phosphorylation levels of LRP6 are increased in *Popdc1* null mutants and are thought to cause an increase in nuclear accumulation of β-catenin. On the other hand, forced expression of POPDC1 in human colon cancer cells was associated with a recruitment of β-catenin to the plasma membrane [44]. A potential mechanism by which POPDC1 regulates cell proliferation could thus entail high POPDC1 expression preventing cytoplasmic accumulation of β-catenin and thereby inhibiting cell proliferation. The suppression of POPDC1 would enable nuclear localization of β-catenin to enhance the transcription of Wnt target genes such as cyclin D1 that regulate the cell cycle resulting in enhanced proliferation and tumorigenesis.

### 5.4. POPDC1 and PP2A

PP2A is one of the major serine-threonine phosphatases that dephosphorylates proteins to regulate multiple signaling pathways in mammalian cells [79]. PP2A is a tumor suppressor that regulates apoptosis and cell cycle progression by counteracting kinase-dependent oncogenic signaling in pathways such as the MEK1 and ERK kinases [79,80].

POPDC1 has been shown to interact with the PP2A-PR61α complex and to promote degradation of the proto-oncogene c-Myc in inflammatory carcinogenesis [10]. c-Myc is a transcription factor that promotes cell proliferation by activating a large number of pro-proliferative genes [79,81]. Consistent with these findings, the loss of POPDC1 also increased c-Myc stability and increased the expression of c-Myc transcriptional targets: Ornithine decarboxylase (Odc) and E2f transcription factor 2 (E2f2) in tumors of Popdc1 null mutant mice [10]. In addition, the forced expression of POPDC1 reduced the stability of c-Myc and increased c-Myc ubiquitylation [10]. The interaction between POPDC1, a tumor suppressor, and one of the main tumor suppressor phosphatases in mammalian cells could thus imply an effective anti-proliferative collaboration that might be relevant in regulating cell proliferation in multiple cell lines and tumors. As mentioned above, the fact that PP2A has also been implicated in the control of LRP6 by POPDC1 [78] could indicate that POPDC1 in general interacts with regulatory PP2A subunits. This interaction could also potentially implicate POPDC1 in pro-apoptotic signaling. However, further studies are required to clarify these mechanisms.

### 5.5. POPDC1 and Bnip3

BCL2 and adenovirus E1B 19-kDa-interacting protein 3 (Bnip3) is a pro-apoptotic mitochondrial membrane-localized protein that belongs to the Bcl2 protein family [82,83]. Bnip3 plays a major role in regulating mitochondrial cell death pathways [82,83]. Mechanisms by which Bnip3 induces cell death include opening mitochondrial permeability transition pores and activation of the apoptosis regulating proteins BAX/BAK [82].

Evidence suggesting that POPDC1 might regulate cell proliferation via a mechanism that involves Bnip3 signaling came from a study that assessed the effects of POPDC1 on cardiomyocyte cell viability. Suppression of POPDC1 in myocytes cultured under serum-starved conditions was recently shown to result in cardiomyocyte cell death and the upregulation of Bnip3 [53]. Furthermore, POPDC1 was shown to regulate Bnip3 expression [53]. Interestingly, this is the first dataset linking suppression of POPDC1 to increased cell death and to mitochondrial-mediated mechanisms of regulating cell viability (Figure 3).

## 6. The Roles of POPDC Proteins Cell Adhesion

The function of POPDC1 in regulating cell adhesion and epithelial integrity have been proposed in various studies [39,61,84]. In contrast, the effects of POPDC2 and POPDC3 on cell adhesion have not been explored. This section will therefore focus on the role of POPDC1 in cell adhesion.

Evidence that POPDC1 is potentially involved in cell adhesion was initially suggested by the observed accumulation of POPDC1 at the points of cell–cell contact in epicardial cells and various other epithelial cell lines [61,85]. Interestingly, the accumulation of POPDC1 only occurred in newly established sites of cell–cell contact [28,61,85]. In dissociated single cells, POPDC1 is mainly localized to the perinuclear region [85] and Golgi apparatus [61], with reduced expression at the cell membrane. Cell–cell contact, however, triggers trafficking of POPDC1 to the cell membrane and enhanced localization at points of cell–cell interaction [85]. The proposed function of POPDC1 as an adhesion protein was based on the erroneous assumption that the carboxy terminus would be extracellular [85]. With only the short amino terminus being localized extracellularly, it is highly unlikely that POPDC proteins are directly involved in cell–cell adhesion. Interestingly, the accumulation of POPDC1 at points of cell–cell contact occurred before the appearance of E-cadherin at the junction [61]. In addition, POPDC1 does not co-localize with E-cadherin at the adherence junctions of epithelial cells. E-cadherin is an essential component of adherence junctions that are involved in the initiation and stabilization of cell–cell adhesion [86,87]. Adherence junctions are formed prior to the assembly of tight junctions in epithelial cells [86]. The accumulation of POPDC1 at points of cell–cell contact before the appearance of E-cadherins thus suggests that POPDC1 potentially plays an essential role in the early processes of establishing cell–cell contact. In addition, the overexpression of POPDC1 into non-adhesive fibroblastic L-cells promoted cell adhesion suggesting that POPDC1 may potentially modulate the expression of proteins that are directly involved in establishing cell–cell adhesion [61,85]. This hypothesis is further supported by data from the same study, showing that the suppression of POPDC1 inhibited epithelial migration and the formation of epithelial sheets [85].

Although POPDC1 does not colocalize with E-cadherin in MDCK epithelial cells [61], POPDC1 has been associated with the regulation of E-cadherin expression in corneal epithelial cells, HCC and colorectal carcinoma cell [7,44,88]. In corneal epithelial cells, the loss of *POPDC1* was associated with reduced expression of E-cadherin [44]. Similarly, the inhibition of *POPDC1* causes a reduction in E-cadherin expression in human HCC [88]. Given the central role of E-cadherins [86,87], the regulation of E-cadherin expression suggests that POPDC1 could be essential in the initiation and maintenance of cell adhesion.

POPDC1 has been shown to co-localize and interact with tight junction molecules, also supporting a role in the maintenance of cell–cell contacts [61]. In epithelial cells, POPDC1 colocalizes with the tight junction proteins ZO-1 and occludin. Analysis of protein interactions using a GST pulldown assay confirmed an interaction between the CDT of POPDC1 and ZO-1, supporting the hypothesis that POPDC1 has a role in the maintenance of cell adhesion [61]. However, POPDC1 did not interact with occludin despite the observed co-localization between the two molecules [61]. Given that POPDC1 specifically interacted with ZO-1 but not with other tight junction proteins suggests that the interaction of POPDC1 and ZO-1 modulates tight junction function. Since the functions of ZO-1 are not limited to tight junction maintenance, the interaction between POPDC1 and the ZO-1 may also affect cell proliferation [61]. As previously discussed, POPDC1 interaction with ZO-1 blocks the transcription of ZONAB/DbpA target genes that regulate cell proliferation (Figure 3A).

Furthermore, POPDC1 has been shown to regulate the integrity of tight junctions. Suppression of POPDC1 causes cell junction disassembly and a loss of transepithelial resistance and reduced epithelial polarization [61]. Re-expression of POPDC1 rescued the knockdown phenotype resulting in increased transepithelial integrity and the retention of ZO-1 at the membrane. Taken together, this data suggests that POPDC1 is potentially essential in the establishment and maintenance of cell adhesion both at adherence and tight junctions. This is also consistent with the hypothesis that POPDC1 is a tumor suppressor in colorectal cancer and hepatocellular carcinoma.

POPDC1 has also been observed to accumulate at the intercalated disk (ID) in cardiac myocytes [89]. However, presently the significance of this observation is unknown. POPDC proteins play a role in mediating cAMP signaling and possibly modulate PKA-dependent phosphorylation. The ID is the site of electromechanical coupling in cardiac myocytes. It is possible that the extent of PKA-dependent phosphorylation of Connexin 43 or SCN5A is modulated by POPDC1. Since phosphorylation of both proteins affect their membrane trafficking, it is plausible to propose that POPDC1 might be involved in the control of electrical coupling between cardiac myocytes [90,91], which represents a testable hypothesis. Recently, it has also been demonstrated that beta-adrenergic signaling strengthens cohesion between cardiac myocytes. It is therefore also possible that cohesion between myocytes (similar to what is proposed for POPDC1 in case of epithelial cells) is controlled by POPDC1 [92]. If POPDC controlled strengthening of cell–cell interactions are also important in skeletal muscle, it would possibly provide an explanation for the defects seen at the MTJ in the embryonic zebrafish tail musculature [14].

## 7. The Roles of POPDC Proteins in Cell Migration, Invasion and Metastasis

Consistent with POPDC1 being a tumor suppressor, the loss of POPDC1 has been shown to promote cell migration, invasion and metastasis in various cancer types [37,38,43,45,88]. Indeed, POPDC1 inhibits cell migration and invasion in hepatocellular carcinoma [38] and colorectal cancer cells [44]. The gain of POPDC1 function also inhibits tumor growth and metastasis of colorectal carcinoma cells [44]. The mechanism by which POPDC1 regulates cell migration, invasion and metastasis can be partly linked to its role in the maintenance of adherence and tight junctions. The loss of cell–cell contact at the tight junctions can lead to the detachment of cells from the primary tumor enabling cells to more easily migrate and invade adjacent tissue or breach the basement [93,94]. These processes represent the initial steps of metastasis that occur prior to the intravasation of tumor cells into blood or lymphatic circulation [93]. The mechanism by which POPDC1 regulates cell migration, invasion and metastasis could therefore be partly linked to its role in the maintenance of adherence and tight junctions.

In vitro experiments using corneal epithelial cells demonstrated enhanced localization of POPDC1 at the cell membrane and adherence junctions in response to cell–cell contact [61,84]. However, in migratory epithelial cells surface expression of POPDC1 was reduced [84]. In addition, the suppression of POPDC1 with the help of antisense morpholino in corneal epithelial cells also resulted in the disruption of epithelial integrity and enhanced cell migration [84]. This is consistent with high POPDC1 expression being required for adhesion maintenance by interacting with tight junction molecules such as ZO-1 [61]. Further support for this hypothesis comes from the fact that high POPDC1 levels reappeared at the epithelial surface when cells ceased to migrate and initiated cell–cell contact [84]. These findings highlight the fact that reduced expression of POPDC1 at the cell surface is favorable to ensure cell migration. In addition, this data corroborates findings in non-adhesive fibroblastic L-cells where the overexpression of POPDC1 in these cells induced adhesive behavior [61,85] suggesting that high POPDC1 expression inhibits cell migration by promoting cell adhesion.

This gives further support to the hypothesis that POPDC1 is a tumor suppressor whose high expression and function inhibits malignant behavior such as the initiation of cell migration, while its loss of function promotes a migratory and malignant phenotype in tumor cells. Interestingly, the suppression of POPDC1 has been shown to promote cell migration and invasiveness of breast cancer cells [37,43], gastric cancer cells [40,45], and hepatocellular carcinoma cells [38,88]. Furthermore, suppression of POPDC1 has been correlated to enhanced metastasis and poor clinical outcomes in gastric cancer [45]. Since loss of adhesion (cell detachment), cell migration and invasion are events that promote metastasis [93,94], the role of POPDC1 in preventing metastasis could be linked to its functions in regulating cell-adhesion.

Although the effects of POPDC2 and POPDC3 on cell migration, invasion and metastasis have not been extensively studied, the suppression of POPDC3 has been shown to stimulate cell migration and invasion in gastric carcinoma cells [45]. In addition, low POPDC3 expression has also been correlated to metastasis and high depth of invasion in gastric cancer [45]. This suggest that POPDC3 potentially regulates cell migration, invasion and metastasis. It is, however, unclear whether POPDC3 regulates these processes via a similar mechanism or potential interaction partners such as POPDC1. Further studies are thus required to elucidate the mechanisms by which POPDC1 and POPDC3 proteins promote cell migration, invasion and metastasis. It would also be interesting to determine if POPDC1 regulates migration via other additional mechanisms or if it interacts with other molecules in addition to ZO-1, that regulate cell adhesion.

POPDC1 has also been shown to interact with molecules in pathways that regulate cell migration such as GEFT, NDRG4 and Netrin-1 [38,54,95]. Hence the effects of POPDC1 on cell migration can potentially also be mediated via its interaction with molecules in these pathways. N-Myc downstream-regulated gene (NDRG4), has been shown to bind the CDT of POPDC1. NDRG4 is a candidate tumor suppressor that is known to modulate cell migration, invasion, proliferation and angiogenesis [95,96,97]. The POPDC1–NDRG4 interaction was further shown to be essential in regulating the directional migration of epicardial cells [95]. In addition, disruptions to the POPDC1-NDRG4 interaction resulted in loss of directional migration and increased cell migration [95]. This suggests that the POPDC1–NDRG4 interaction is essential in controlling the direction and rate of migration in these cells.

In a similar fashion, the CTD domain of POPDC1 has been shown to interact with the guanine nucleotide exchange factor (GEFT) [54]. GEFT regulates the active state of GTPases such as Rac1 and RhoA which are known to regulate cell migration [98,99,100]. As previously discussed, the POPDC1–GEFT interaction is thought to potentially control migration via regulation of the activity of GTPases such as Rac1 and Cdc42. The POPDC–GEFT interaction could therefore represent another novel mechanism by which POPDC1 regulates cell migration.

Lastly, POPDC1 has also been implicated in the regulation of netrin-1-mediated cell migration and invasion. Netrin-1 belongs to the netrin family of extracellular proteins that guides the migration of cells and axons [101,102]. In cancer, netrin proteins regulate cell adhesion, migration, and survival [101]. In HCC, the expression of netrin-1 negatively correlates with *POPDC1* expression [38]. The overexpression of netrin-1 also suppressed POPDC1 expression in these cells suggesting that *POPDC1* is potentially regulated by netrin-1 in HCC. Given that both POPDC1 and netrin-1 are known to regulate migration and invasion, the effects of POPDC1 on netrin-1 mediated cell migration and invasion was tested. Interestingly, the upregulation of POPDC1 in HCC attenuated the ability of netrin-1 to enhance cell migration and invasion. This suggests that netrin-1 potentially enhances cell migration and invasion via a mechanism that entails POPDC1 suppression.

Taken together POPDC1 and POPDC3 regulate cell migration, invasion and metastasis. While various mechanisms by which POPDC1 potentially controls migration are known, further studies are warranted to clearly elucidate these mechanisms. Investigating the roles and mechanisms by which POPDC2 and POPDC3 potentially regulate migration, invasion and metastasis will also provide the much-needed clarity on how diverse the functions of POPDC proteins might be in various cancer types. Clarifying these mechanisms is thus essential to inform strategies on how these proteins can best be targeted in the treatment of pathologies such as cancer.

## 8. Working Model of POPDC1 as A Tumor Suppressor

We postulate that the function of POPDC1 as a tumor suppressor is primarily dependent on its proper plasma membrane localization. In normal physiology, high levels of POPDC1 are localized at the cell membrane where it interacts with tight junction proteins such as ZO-1 to maintain epithelial integrity and cell–cell contact. High expression of ZO-1 at the tight junctions also results in low nuclear expression of ZONAB/DbpA, causing reduced activation of ZONAB/DbpA-regulated cell cycle gene [44,66,68]. This would result in the simultaneous inhibition of cell adhesion, migration and proliferation (Figure 3).

In cancer, the tumor suppressor functions of POPDC1 are reduced due to protein suppression and mislocalization of the protein to the cytoplasm and nuclear membrane. This results in low POPDC1 expression at the cell membrane which reduces its interaction with tight junction molecules, leading to a loss of adhesion and cell–cell contact. Loss of cell adhesion will induce a migratory phenotype which consequently increases cell migration, invasion and metastasis. Since the interaction between POPDC1 and ZO-1 is thought to increase ZONAB/DbpA accumulation at the tight junction, the mislocalization of POPDC1 to intracellular compartments would reduce its interaction with ZO-1 leading to the accumulation of ZONAB/DbpA at the nuclear membrane. This would result in increased transcription of ZONAB/DbpA target cell cycle genes such as HER2, cyclins D1 and PCNA, promoting cell proliferation (Figure 3). We also hypothesize that POPDC1 potentially regulates other transcription factors. The accumulation of POPDC1 at the nuclear membrane (and the nucleoplasm) can thus potentially increase cell proliferation by increasing the activity of transcription factors that are regulated by POPDC1.

## 9. Outlook

It is evident that POPDC genes serve an important homeostatic function in many different organs and cell types. It is very likely that the common functions of POPDC proteins in striated muscle and epithelial cell biology are related to their role as cyclic nucleotide effector proteins given that this part of the protein is shared by all three isoforms and is the most evolutionary conserved part of the protein. Research has significantly progressed in defining POPDC functions in striated muscle and epithelial tissues. However, our understanding of the precise role of POPDC proteins is still lagging behind that of EPAC and PKA. This is for, example, partly due to the lack of a crystallography- or NMR-derived structure of the Popeye domain. Such a structure would help to identify the allosteric changes induced by cAMP binding, something that has been well-studied in the case of PKA and EPAC proteins [6,103]. Moreover, a Popeye domain structure would also facilitate the identification of agonists and antagonists that specifically modulate POPDC protein function and ideally these compounds will need to be isoform-specific in order to define the unique roles of each of the POPDC isoforms. Likewise, a Popeye domain structure would also be helpful in designing Popeye domain-based sensors to monitor cAMP binding to the Popeye domain in response to the stimulation of various GPCRs. Popeye domain-based cAMP sensors would help to define cAMP nanodomains that are dependent on POPDC protein function [104,105]. It is also of great interest to establish whether POPDC proteins do interact with other proteins of the cAMP signaling pathway. In the case of EPAC and PKA, compartmentalized activation is achieved through interaction with A-kinase-anchoring proteins [106]. It is presently not known whether POPDC proteins form protein complexes with EPAC or PKA. It is also possible that POPDC proteins serve a function as AKAP-like proteins, which are able to sense cAMP levels and nucleotide binding, and could modulate the protein complex that is bound to them. AKAP proteins are protein binding platforms and assemble site-specific signalosomes [107]. They bind GPCRs, ACs and PDEs along with PKA, and several protein substrates, which are phosphorylated in response to GPCR stimulation. The precise makeup of these complexes allows high-fidelity spatiotemporal control of the cellular response in a receptor-specific manner [104,105]. It is possible that POPDC proteins also assemble cAMP signaling complexes. In order to address this question, we will need to make progress in defining how POPDC proteins may have an impact on any of the increasing numbers of interacting proteins. A well-established effect of POPDC proteins is the modulation of membrane trafficking of interacting proteins, which is well-documented in case of TREK-1 and POPDC2 proteins model [14,19]. However, we do not precisely know how POPDC proteins are able to modulate protein trafficking and how they engage with the endosome-based protein trafficking machinery [108].

It is evident, that a lot more research is required to define the biological functions of POPDC proteins in striated muscle and epithelial cells. A better understanding of their role will undoubtedly have a strong impact on cardiovascular medicine, myology and tumor biology.

## Figures and Tables

**Table 1:** Cardiac and skeletal muscle phenotypes in model organism and patients.

Species	Mutation	Heart	Skeletal Muscle	References
**mouse**	*Popdc1^−/−^*	stress-induced sinus bradycardia	regeneration defect	[19,27]
		ischemia-reperfusion damage		[28]
	*Popdc2^−/−^*	stress-induced sinus bradycardia	no phenotype reported	[19]
**zebrafish**	*popdc1* morphants	AV-block, pericardial effusion	muscular dystrophy	[14]
	*popdc2* morphants	AV-block, pericardial effusion	muscular dystrophy	[30]
	*popdc1* ^S191F^	AV-block, pericardial effusion	muscular dystrophy	[14]
**human**	*POPDC1*			
	p.S201F	2nd degree AV-block	LGMDR25	[14]
	c.1A > G	1st degree AV-block	LGMDR25	[23]
	p.V217-L272del	1st/2nd degree heart block	LGMDR25	[23]
	p.R88X	1st degree AV-block	LGMDR25	[23]
	*POPDC2*			
	p.W188X	3rd degree AV-block	no muscle phenotype	[31]
	*POPDC3*			
	p.L155H	no cardiac phenotype	LGMD	[17]
	p.L217F	no cardiac phenotype	LGMD	[17]
	p.R261Q	no cardiac phenotype	LGMD	[17]
